# Iron Status and Febrile Seizure- A Case Control Study in Children Less Than 3 Years

**Published:** 2012

**Authors:** Mansour SADEGHZADEH, Parisa KHOSHNEVIS ASL, Esrafil MAHBOUBI

**Affiliations:** 1 Assistant Professor of Pediatric, Department of Pediatrics, Zanjan University of Medical Sciences, Zanjan, Iran; 2 Pediatrician, Zanjan University of Medical Sciences, Zanjan, Iran

**Keywords:** Seizures, Febrile, Anemia, Iron deficiency

## Abstract

**Objective:**

Febrile seizure is one of the most common neurological conditions of childhood. Several theories, such as iron deficiency anemia have been proposed as the pathogenesis of this condition. The aim of this study was to find the association between iron deficiency anemia and febrile seizures in children aged 6 months to 3 years admitted in Valie Asr hospital in Zanjan.

**Materials &Methods:**

Hemoglobin (Hb), mean corpuscular volume (MCV), serum iron (SI), total iron binding capacity (TIBC) and SI/TIBC ratio were assessed in one hundred children with febrile seizures and compared to the values of one hundred healthy children presenting in a heath care center in the same period as the control group.

**Results:**

A total of 6% of cases had iron deficiency anemia which was similar to the control group. In the case group SI/TIBC ratio below 12% was seen in 58% of children which was significantly higher than that of the control group (29%).

**Conclusion:**

The results of this study suggest that although anemia was not common among febrile seizure patients, iron deficiency was more frequent in these patients.

## Introduction

Febrile seizure (FS) is one of the most common neurological conditions of childhood with an incidence of about 2-14% in different societies (1-2). Several theories, such as genetic factors have been proposed as the pathogenesis of this condition (1). Although the role of micronutrients such as zinc (2) and iron (3-5) have been largely studied as the predisposing factors, there is still a need to explore their relation to febrile seizures (6).

Iron plays an important role in brain energy metabolism (1,7), myelin formation and neurotransmitter metabolism (3, 8-10). Iron deficiency affects the regional monoamine metabolism, such as serotonin, dopamine and norepinephrine, glutamate and gamma-aminobutyric acid (GABA) (11-14).The fetal brain may be at risk even if the infant is not anemic, because when there is not enough iron supply, the first priority of iron is red blood cells instead of other tissues(15).

It has also been suggested that iron deficiency lowers the seizure threshold and increases the risk of febrile seizures (3,16). The relationship between iron deficiency anemia and FS has been evaluated in several studies with conflicting results (1, 6, 17,18).

Considering the prevalence of iron deficiency anemia in Iranian children (estimated about 20%) (19-20) and the frequency of febrile seizure in our region, we conducted a case control study to evaluate the association between iron deficiency anemia and febrile seizure in children in Zanjan, northwest of Iran.

## Materials & Methods

In this prospective case control study, 100 children aged 6 months to 3 years with the diagnosis of febrile seizures admitted at Valie Asr hospital in Zanjan were enrolled. The control group consisted of randomly 100 age-matched children referring to a heath care center for routine growth assessment. The process was completely explained and an informed consent was obtained from the parents.

After admission, all cases were completely examined to exclude children with a previous history of epilepsy, developmental delay, neurologic deficit and CNS infection. Information on age, gender, body temperature upon admission, cause of fever, duration between initiation of fever and convulsion, family history of febrile convulsion and details of the seizure history including duration, frequency and type of seizure (simple or complex) were recorded for all cases and controls (if applicable) in a questionnaire. Tonicclonic or tonic seizures lasting for less than 15 minutes without focal signs with a short postictal period were defined as simple; whereas, seizures of more than 15 minute-duration occurring more than once in 24 hours or focal features were considered complex.

Blood samples were collected to measure hemoglobin (Hb), serum iron (Fe), total iron binding capacity (TIBC) and mean corpuscular volume (MCV). Iron deficiency anemia was defined as a hemoglobin value less than 10.5g% and the serum iron to TIBC ratio less than 12% and MCV less than 70fl (1,4); whereas, iron deficiency was considered as a serum iron to TIBC ratio less than 12%. The Ethics Committee of Zanjan University of Medical Sciences (ZUMS) approved the study.

Discrete variables are expressed as counts (%) and compared using the Chi-square tests. Statistical analysis was performed by independent t test and Pearson correlations using SPSS 16.0 software (SPSS Inc., Chicago, Illinois, USA). Differences were considered statistically significant if the P value was less than 0.05.

## Results

In the case group, there were 60 (60%) male and 40 (40%) female. The control group consisted of 59 (59%) boys and 41 (41%) girls. Febrile seizure was most commonly seen in the 12-23 months age group (53%). 

In 20% of the cases, there was a positive family history of febrile convulsion. Thirty children (11 female, 19 male) had complex febrile seizure. Twenty nine patients had recurrent seizures and in one patient the duration of seizure was more than 15 minutes. Three patients had recurrent seizure lasting more than 15 minutes. Complex seizures as well as simple febrile seizures were more frequent in males and in 12-23-months old patients. The distribution of simple and complex seizure among different age groups is shown in Table 1. Data on the patients’ hematologic status is shown in Table 2. As shown in the table, hemoglobin less than 10.5gr%, MCV less than 70fl and serum iron less than 30mg% were higher among the cases as compared to the control group, but their differences were not statistically significant (p=0.47, p=0.2 and p=0.65, respectively).

Serum iron to TIBC ratio less than 12% was found in 58% of the cases (63.3% of patients with complex febrile seizure and 57.1% of patients with simple febrile seizure) which was significantly higher than the controls (p=0.000035).

We found iron deficiency anemia, defined as a hemoglobin value less than 10.5g% and a serum iron to TIBC ratio less than 12% and an MCV less than 70fl, in 6% of both control and case groups.

## Discussion

In our study, although the level of Hb, serum iron and MCV were not statistically different in cases compared to the reference group, the serum iron to TIBC ratio were significantly lower in patients. There is a controversy regarding the role of ‘‘iron status’’ in febrile convulsion (1, 16). Some studies have shown that iron deficiency is more common in patients with febrile seizure and have concluded that it could be considered as a risk factor of febrile seizure (3,4,6,21-26); whereas, in some other studies, iron deficiency did not have a predisposing role in febrile seizure (1,16,17,27) and it even had a protective effect (28,29). Iron deficiency anemia was noted in 6% of our patients, which is similar to the study conducted by Hartfield (21). This figure was 44% in Bidabadi (1), 63.6% in Kumari (3), 30% in Pisacane (4), 31.8% in Sherjil (6), 29% in Momen (18) and 42% in Abbaskhanian (29) et al. studies.

In our study, iron deficiency anemia was similar in both case and control groups. These results are similar to reported studies by Bidabadi (1), Momen (18), Daoud (22), and Amirsalari (27), but are different from the studies carried out by Kumari (3), Pisacane (4), Sherjil (6) and Naveed-ur-Rehman (23) which showed a significantly higher frequency of iron deficiency anemia in patients with febrile seizures. 

Kobrinsky (28) and Abbaskhanian (29) et al. found a higher level of iron deficiency anemia in the control groups and concluded that anemia raises the threshold for febrile seizure and iron deficiency may protect against the development of febrile convulsions. 

The results of this study demonstrated the significantly higher frequency of iron deficiency in patients with febrile seizure. The same results are shown by Kumari (3), Hartfield (21), Daoud (22) and Vaswani (24) et al., but the results by Bidabadi (1), Salehi (17), Amirsalari (27) and Idro (16) et al. are different. 

As mentioned above, there are contradictory results regarding the role of iron deficiency and iron deficiency anemia in febrile seizures. The authors believe that differences between the results of this study and other studies may be due to the use of different definitions for iron deficiency and iron deficiency anemia, variability of the control groups and the sample size, different etiologies for fever and the use of premedication. 

**Frequency**

**Table 1:** Distribution of Simple and Complex Febrile Seizures in Different Age Groups

**Age group (months)**	**Type of Seizure**	**Frequency** ** (Percentage)**	**Frequency** ** (Percentage) In total**
**6-11**	Simple febrile seizures	19(27.4%)	19(19%)
	Complex febrile seizures	3(10%)	3(3%)
**12-23**	Simple febrile seizures	36(51.4%)	36(36%)
	Complex febrile seizures	17(56.7%)	17(17%)
**24-36**	Simple febrile seizures	15(21.4%)	15(15%)
	Complex febrile seizures	10(33.3%)	10(10%)
**Total**	Simple febrile seizures	70(100%)	70(70%)
	Complex febrile seizures	30(100%)	30(30%)

**Table 2:** Distribution of Different Variables in Cases and Controls

	Group	**Frequency**	**Percentage**	**P Value**
**Hb<10.5 g/dl**	Febrile seizure	21	%21	0.47
	Control	17	%17	
**Hb>10.5 g/dl**	Febrile seizure	79	%79	0.47
	Control	83	%83	
**MCV<70 fl**	Febrile seizure	15	%15	0.2
	Control	22	%22	
**MCV>70 fl**	Febrile seizure	85	%85	0.2
	Control	78	%78	
**Fe <30 mg/dl**	Febrile seizure	12	%12	0.65
	Control	10	%10	
**Fe >30 mg/dl**	Febrile seizure	88	%88	0.65
	Control	90	%90	
**Fe/TIBC<12%**	Febrile seizure	58	%58	0.000035
	Control	29	%29	
**Fe/TIBC>12%**	Febrile seizure	42	%42	0.000035
	Control	71	%71	

**In conclusion, **the results of this study suggest that although anemia was not common among febrile seizure patients, iron deficiency was more frequent in these patients. 

We suggest a prospective case control follow up study in larger samples with treatment of all iron deficient and anemic patients and estimating the effect of treatment on the recurrence of febrile seizures.
